# Deciphering the Roles of Innate Lymphoid Cells in Cancer

**DOI:** 10.3389/fimmu.2019.00656

**Published:** 2019-04-03

**Authors:** Melanie Bruchard, Francois Ghiringhelli

**Affiliations:** ^1^INSERM UMR1231, Dijon, France; ^2^University of Burgundy and Franche-Comté, Dijon, France

**Keywords:** cancer, immunotherapy, innate lymphoid cells, MDSC, cytokine

## Abstract

Cancer is a complex disease and the role played by innate lymphoid cells (ILCs) in cancer development has begun to be uncovered over recent years. We aim to provide an exhaustive summary of the knowledge acquired on the role of ILCs in cancer. ILCs are classified into 3 different categories, ILC1s, ILC2s, and ILC3s, each encompassing specific and unique functions. ILC1s exhibit NK cells characteristics and can exert anti-tumor functions, but surprisingly their IFNγ production is not associated with a better immune response. In response to TGF-β or IL-12, ILC1s were shown to exert pro-tumor functions and to favor tumor growth. ILC2s role in cancer immune response is dependent on cytokine context. The production of IL-13 by ILC2s is associated with a negative outcome in cancer. ILC2s can also produce IL-5, leading to eosinophil activation and an increased anti-tumor immune response in lung cancer. ILC3s produce IL-22, which could promote tumor growth. In contrast, ILC3s recognize tumor cells and facilitate leukocyte tumor entry, increasing anti-tumor immunity. In some contexts, ILC3s were found at the edge of tertiary lymphoid structures, associated with a good prognostic. We are at the dawn of our understanding of ILCs role in cancer. This review aims to thoroughly analyze existing data and to provide a comprehensive overview of our present knowledge on the impact of ILCs in cancer.

## Introduction

Innate lymphoid cells (ILCs) are a heterogeneous immune cell population identified in the late 2000's by several teams (1–8). Previously known lymphoid tissue inducer (LTI) cells and natural killer (NK) cells, discovered in 1997 and 1975, respectively, are also members of the ILC family (9, 10). ILCs lack antigen specific receptors and their development is independent from rearrangement genes. ILCs mirror T lymphocytes subpopulations in their expression of master regulator transcription factors and cytokine production; they are therefore considered their innate counterparts (11). ILCs are amongst the first responders when facing a threat and they help shaping both innate and adaptive immune responses thanks to cytokine production. ILCs are mostly tissue resident where they seed during fetal life (12). ILCs begin functioning during fetal development (13).

ILCs are divided into 3 main groups, ILC1s, ILC2s, and ILC3s, depending on the expression of transcription factors and cytokines (11). ILC1s and ILC3s contain several subgroups members with different functions. Group 1 ILCs comprise NK cells, ILC1s and recently described intra-epithelial ILC1s as well as ILC1 like cells (14, 15). Group 3 ILCs gather LTI cells, Natural Cytotoxicity Receptor (NCR)+ ILC3s and NCR- ILC3s, with some NCR- ILC3s expressing CCR6 (16). ILC1s, ILC2s, and ILC3s constitute the innate equivalents of CD4+ T helper Th1, Th2, and Th17, respectively, while NK cells are the counterpart for cytotoxic CD8 T cells.

Group 1 ILC, like Th1, are regulated by Tbet and can produce Interferon γ (IFNγ), GM-CSF (Granulocyte-Macrophage Colony-Stimulating-Factor), granzyme and perforin in response to IL-12, IL-18 or other activators. They cooperate with Th1 cells against intracellular microbes such as virus, bacteria or parasites. Group 1 ILCs activate macrophages and some can exert direct cytotoxicity (17, 18). Group 2 ILCs, similarly to Th2, express Gata3 and can produce IL-4, IL-5, IL-13, IL-9, and amphiregulin in response to IL-25, IL-33, and TSLP (Thymic Stromal LymphoPoietin). Group 2 ILCs are essential in the immune response against parasites and allergens and their production of amphiregulin promotes tissue damage repair (5, 19). Group 3 ILCs, mirroring Th17, express RORγt, the lymphotoxins α and β, IL-17 and IL-22, GM-CSF, and Tumor Necrosis Factor α (TNFα). They can be activated by IL-23, IL-1β, or by NCR ligands and they are involved in the immune response against extracellular microbes such as fungi or bacteria (1, 3, 20). LTI, members of group 3 ILCs, are essential for the formation of secondary lymphoid structures during embryonic development (10). The Fan team recently identified a fourth group of ILCs, both in mouse and human, called ILCregs (21). These ILCregs can be induced during inflammation in the intestine where they control ILC1 and ILC3 activation as well as cytokine production thanks to their IL-10 production, preventing intestinal inflammatory associated damage. ILCregs also produce Transforming Growth Factor (TGF)-β that sustains their maintenance and expansion in a paracrine manner.

CD4 T lymphocytes and ILCs have some redundant as well as specific functions. Phylogenetic studies have suggested that ILCs probably appeared latter in evolution than T and B lymphocytes (22). They appeared in vertebrates and the ILCs complete family can only be found in mammals. This finding probably means that ILCs do not constitute a simplified version of adaptive immune system but rather a complementary one.

The plastic nature of ILCs is well documented. Like T cells, ILCs adapt phenotype and functions upon the polarizing signals they are exposed to. Evidence of this plasticity was first seen between groups with the conversion of ILC3s into ILC1s (17, 23–25). ILC2s can also convert into IFNγ producing ILC1s (13, 26, 27). Furthermore, ILCs can also evolve while remaining within the same group, an example are NK cells that become intermediate ILC1 cells upon TGF-β stimulation (28, 29). These intermediate ILC1s present a phenotype with characteristics of both ILC1 and NK cells, and are identified as CD49a+ and Cd49b+.

Another challenge in ILCs study is the heterogeneity of the surface markers found within each subgroup depending on tissue localization or disease. The expression of CD69 in ILC2s is restricted to skin, spleen and mucosal tissue while CD69 can be found together with ICOS (Inducible T-cell COStimulator) on NKP44- ILC3s from the spleen and mucosal tissues. Within pathological tissues, ICOS and CD69 are found upregulated on both ILC2s and ILC3s (15). Discrepancies in the markers used to identify ILCs can lead to study slightly different cells under the same name, increasing data variability. Some studies exclude CD5, while others have used this marker to identify functionally immature ILCs in humans (30). Differences on the markers chosen to identify ILCs and expressed by ILCs under specific conditions should be kept in mind, in particular in pathological situations.

Over the years, ILCs were shown to have a massive impact in many diseases like asthma and allergy, multiple sclerosis or inflammatory bowel disease. This review will focus on the role of ILC1s, ILC2s, and ILC3s in cancer, regardless of NK cells. In solid tumors, infiltrating immune cells are needed to trigger an efficient immune response. Tissue resident ILCs present at the tumor site are ideally located to tune anti-tumor immunity. Studies have shown that ILC frequencies vary in cancer. A higher frequency of ILC1s was observed in gastrointestinal tumors whereas an increased frequency of ILC2s was observed in breast and gastric cancer (31, 32). Tumor infiltrating ILCs can show an activated phenotype (expression of CD69, CD44, MHCII, and KLRG1) (32). The impact of NK cells in anti-tumor immune response has been thoroughly documented and numerous reviews are available, therefore this topic will not be addressed here. Despite increasing knowledge on the role of ILCs in cancer cell biology, our understanding on ILCs impact on anti-tumor immune responses remains an emerging field. Previous reviews have focused on the roles of ILCs in cancer (33), but this review aims at analyzing the many progresses made in the recent years.

## ILC1s

A multitude of studies demonstrated an increased proportion of traditional ILC1s in cancer. ILC1s percentage increase was found in peripheral blood mononuclear cells (PBMC) of untreated acute myeloid leukemia patients with reduced cytokine production (Table 1) (35). A similar observation was done in PBMC from patients with chronic lymphocytic leukemia (36) but no available studies have elucidated the consequences of ILC1s increase in the efficacy of anti-tumor response or on patients' outcome.

**Table 1:** ILC1 phenotypes and functions in solid and hematological cancers.

	**Organism**	**Cancer**	**Effect against cancer**	**Phenotype**	**Responsive to**	**Production**	**Observations**	**References**
ILC1	Human	Acute myeloid leukemia	ND	Lin- CD127+ CD117– CRTH2–NKp46–		Diminished production of IFNγ and TNFα	Increase in ILC1 % in PBMC	(34, 35)
	Human	Chronic lymphocytic leukemia	ND	Lin– CD127+ CD161+ CRTH2– CD117–		Reduced production of TNFα compared to healthy control	Increase in ILC1 % in PBMC	(36)
Intraepithelial ILC1	Human	Crohn's disease	ND	CD49a+ NKp44+ CD103+ CCR6– CD127– CD56+	IL-15 and IL-12	Production of IFNγ and Granzyme b	Increase in ILC1 % in the epithelium	(18)
Intraepithelial ILC1	Mouse	CD40 colitis	–	CD160+NKp46+NK1.1+CD3–	IL-15	Production of IFNγ and Granzyme b	Increase in ILC1 % in the epithelium and contribution to intestinal inflammation	(18)
Type 1 like ILC	Mouse	Mammary pre-cancerous lesions	+	CD127– CD49a+ TCR– NK1,1+	IL-15	Production of Granzyme b	Increase in ILC1 % in tumor	(14)
Intermediate ILC1	Mouse	MCA1956	–	CD49a+ b+ Lin– CD45+ NK1.1+ NKp46+ Eomes+	TGF-β	Production of GM-CSF and TNFα and decrease in IFNγ and CCL5	Increase in ILC1 % in tumor	(29)

*ND, not determined; PBMC, peripheral blood mononuclear cells*.

ILC1s can be activated by various cytokines like IL-12, IL-15 (18), IL-18 (17), or TGF-b (29) or by viruses (37) and intracellular bacteria (38). In cancers triggered by viruses (e.g., cervical cancer) or by bacteria (e.g., stomach cancer), we would expect an activation of ILC1s, the production of IFNγ and TNFα, leading to anti-tumor immune responses. However, we will see that ILC1s in cancer display a more complex behavior.

Th1 cells are known for their anti-tumor capabilities (39) and type 1 ILCs were also shown to have anti-tumor properties in an IL-15 rich environment. Type 1 ILCs are characterized by particular surface markers such as CD127- and CD49a+ and by their ability to produce granzyme B. These type 1 ILCs expanded in mouse mammary pre-cancerous lesions and they exhibited potent cytotoxic activities against tumor cells, limiting tumor growth. Like NK cells, type 1 ILCs, are dependent on IL-15. Nevertheless, they showed downregulation of signature NK genes while expressing high levels of ILC1 signature genes (14).

Intraepithelial ILC1s are an ILC1s subtype identified in the colon. Like type 1 ILCs, these cells also express CD49a and are responsive to IL-15. After IL-12 stimulation, intraepithelial ILC1s produced IFNγ and granzyme B. However, these intraepithelial ILC1s were shown to expand in patients' colon during Crohn's disease and to contribute to pathology development in a mouse model of CD40 induced colitis (18). The role of these ILC1s in colorectal cancer (CRC) remains to be determined. As patients with ulcerative colitis are at higher risk of developing CRC, it is likely ILC1s might maintain a pro-inflammatory environment and favor cancer development (40).

Studies on TGF-β recently shed light on pro-tumor functions of ILC1s. TGF-β, a cytokine often found in solid cancers, is known to limit tumor immunosurveillance through its action in many cell types (41). Recent data showed that TGF-β could induce NK cells conversion into intermediate ILC1s (29). These cells are CD49a and CD49b double positive and arise in TGF-β rich tumor while NK cells numbers drop. Unlike the NK cells they originated from, which show anti-tumor properties, these intermediate ILC1s were unable to control local tumor growth or metastasis development. They expressed higher levels of the inhibitory immunological checkpoint receptor CTLA-4 (cytotoxic T-lymphocyte–associated antigen 4), Lag3 and CD96 and of the myeloid growth factor GM-CSF. Moreover, these cells expressed low levels of IFNγ and CCL5, a chemoattractant and Th1 activator. All these factors contributed to the establishment of a pro-tumor microenvironment. Furthermore, tumor immune escape was also shown to be partially due to ILC1s production of TNFα (29). Another team further investigated the shift from NK cells into ILC1 like cells in a TGF-β rich environment. They observed that SMAD4 was essential to prevent NK cells from becoming ILC1s when exposed to TGF-β. SMAD4 restrained non-canonical TGF-β signaling and prevented NK cells from developing ILC1 features. Moreover, it also preserved anti-tumor functions and proliferative properties of these cells. NK cells deficient in SMAD4 acquired an ILC1 like gene signature and were unable to control tumor growth and metastasis or infections (28). Interestingly, TGF-β signaling via the TGFR2 in salivary gland ILC1s enhanced IFNγ and Eomesodermin (EOMES) expression while decreasing CD49a expression (42), proving that the presence of one cytokine is not sufficient to dictate the ILC1 response.

## ILC2s

Clinical studies noted an increase in ILC2s in breast (32), gastric (31), or prostate cancer (35). Like Th2, their adaptive counterparts, ILC2s produce IL-4 and IL-13, which are known to participate in the establishment of a pro-tumor microenvironment (43). IL-4 and IL-13 could be used as a predictor of clinical outcome for clear-cell renal cell carcinoma, with low levels of cytokines being linked with a better outcome (44). ILC2s can also produce amphiregulin, which, on one hand, helps tissue repair and controls local inflammation via Tregs but, on the other hand, promotes the growth of tumors expressing the epidermal growth factor receptor (EGFR), tissus invasion and metastasis (45) and enhances Tregs functions and thus immunosuppression (46). Consequently, ILC2s like Th2, are considered pro-tumoral and a potential target to improve anti-tumor immune responses (Table 2).

**Table 2:** ILC2 phenotypes, functions, and associated cells in solid and hematological cancers.

	**Organism**	**Cancer**	**Effect against cancer**	**Phenotype**	**Responsive to**	**Production**	**Associated cell**	**Observations**	**References**
ILC2	Human	Breast	ND	CD45+ Lin– CD56– CRTH2+ CD127+ CD117+/–		Activated phenotype MHCII+ KLRG2+ CD69+ CD44+		Accumulation of ILC2s in tumor tissue displaying increased expression of PD1 and CTLA4 compared to PBMC ILC2s	(32)
	Human	NSCLC	ND	Lin– CRTH2+ CD127+				Lower ILC % in tumor	(47)
	Human	Gastric	ND	Lin– ICOS+ IL-17rb+			M-MDSC	Increased frequency of ILC2s and MDSCs in PBMC	(31)
	Human	Prostate	ND	Lin– CD127+ CRTH2+ NKp46–			M-MDSC	Increased frequencies of ILC2s and M-MDSC in tumor associated with stage of cancer	(35)
	Human	Acute promyelocytic leukemia	–	Lin– CD127+ CRTH2+ NKp46–	NKp30-B7H6 interaction	Increase in IL-13	M-MDSC	Increase in ILC2 % in PBMC	(35)
	Human	Bladder	–	Lin– CD127+ ST2+ NKp46–	BCG and cancer cells	Production of IL-13	M-MDSC	Recruit and induce M-MDSC in tumor	(48)
	Mouse	APL B6	–	Lin– CD127+ ST2+ NKp46–	PGD2 and B7H6	Production of IL-13	M-MDSC	Increase M-MDSC population	(35)
	Mouse	4T1 mammary cancer	–	Lin– Sca-1+ CD25+ CD44+ ST2+	IL-33		M-MDSC and Treg	Increase in ILC2 % in tumor and can reduce NK cytotoxicity	(49)
	Mouse	B16 melanoma	–	CD25+CD45+CD90+CD127+ST2+	IL-33	Expression of CD73		Expansion and activation of ILC2s that can inhibit NK cell activation and cytotoxicity	(50)
	Mouse	TAA CCL4 hepatic fibrosis	–	Lin– CD45+ IL-33R+ IL-7Rα+ CD44+ CD90.2+ ICOS+ Sca1+	IL-33	Production of IL-13		Expansion and activation of ILC2s that can sustain fibrosis	(51)
	Mouse	Egg-associated pulmonary fibrosis	–	Lin– IL-7Rα+ Sca-1+ T1/ST2+ ICOS+	IL-25	Production of IL-13		Activation of ILC2s that are responsible for pulmonary collagen deposition	(52)
	Mouse	Lymphoma	+	Lin– Sca-1+ CD117+ IL-7Rα+	IL-33	Secretion of antitumor CXCL1 and CXCL2		Expansion of ILCs population	(53)
	Mouse	B16 melanoma in lung	+	Lin– Sca-1+ Thy1.2+ CD25+ CD27+ CD44+ CD69+ IL-7Rα+ T1/ST2+	IL-33 and IL-25	Production of IL-5		Eosinophils recruitment	(54)

*ND, not determined; APL, acute promyelocytic leukemia; TAA, thioacetamide hepatic fibrosis; CCL4, carbontetrachloride haptic fibrosis; NSCLC, non-small cell lung carcinoma; M-MDSC, monocyte-myeloid derived suppressor cells; PBMC, peripheral blood mononuclear cells*.

In a clinical study published in 2017, patients in remission with an acute promyelocytic leukemia showed a decrease in Prostaglandin D2 (PGD2) and IL-13 production. ILC2s and monocytic myeloid derived suppressor cells (M-MDSC) cell numbers were also reduced (35). M-MDSC is an important immunosuppressive cell population (55). In mice, ILC2s are expanded and activated through the interaction of CRTH2 and NKp30 with tumor derived PGD2 and B7H6, respectively, leading to an increased production of IL-13. M-MDSC, which express the IL-13 receptor alpha1 are in turn activated by ILC2s produced IL-13 and set up a pro-tumor environment (48). Blocking PGD2, IL-13, and NKp30 partially reduces the levels of ILC2s and M-MDSC in PBMC and leads to increased survival in animal models (35). A positive correlation was also found between ILC2s and M-MDSC in patients bearing prostate and bladder cancer (35, 48). This ILC2/M-MDSC immunosuppressive axis was shown to be dependent on the T/M-MDSC ratio. Patients with a ratio of more than 1 (less M-MDSC than T cells) showed a dramatically higher recurrence free survival than patients with more M-MDSC than T cells in the context of bladder cancer (48).

IL-33 activates ILC2s. This cytokine is found in mouse and human breast cancers (56) and colorectal cancers (57) and could potentially directly activate ILC2s. In the 4T1 mouse mammary cancer model, IL-33 was associated with an increase in M-MDSC, ILC2s, and Tregs. IL-33 also accelerated tumor growth and metastasis development, by sustaining the ILC2/M-MDSC/Tregs immunosuppressive axis as well as by promoting neovascularization (49). In the B16 melanoma model, it was also recently shown that IL-33 induced NK cells mediated anti-tumor immune responses in parallel with the expansion and activation of ILC2s via its receptor ST2. ILC2s expression of the ectonucleotidase CD73 (capable of transforming ATP into immunosuppressive adenosine along with CD39) inhibited the activation and cytotoxicity of NK cells, resulting in a less efficient tumor growth control (50). This study highlights that one cytokine can trigger opposite reactions as well as a new suppressive ILC2s mechanism via CD73 expression. The link between IL-33/ILC2s/Tregs was also observed in an allergic setting in lungs (58). In this context, IL-33 induced the expression of OX-40L on the surface of ILC2s, which was shown to be essential for Th2 and Tregs responses. The broad expression of IL-33 in cancers and the pro-tumor functions of Th2 and Tregs suggest ILC2s may facilitate the tumor growth by helping the installation of a pro-tumor immunity.

ILC2s produce amphiregulin, a molecule that orchestrates tissue homeostasis and wound repair. If uncontrolled, this protein can contribute to pathological fibrosis in the context of chronic inflammation. Pathological fibrosis and tumorigenesis can both result from a prolonged and exacerbated healing response. In the liver, fibrosis will progress to cirrhosis and finally to hepatocellular carcinoma, which is why the study of fibrosis development can bring meaningful insights to the development of cancer (59). It was demonstrated that IL-13 potentiated collagen deposition and fibrosis in lung, liver and intestine (46). A study reported that IL-33 was required and sufficient for severe hepatic fibrosis *in vivo*. The pro-fibrotic effect of IL-33 was ILC2 dependent. In thioacetamide (TAA) and carbontetrachloride (CCL4) models of hepatic fibrosis, increased IL-33 production favored the expansion and activated liver resident ILC2s. Depletion of ILC2s or IL-13 were sufficient to limit fibrosis development (51). Accordingly, another study identified IL-25 activated ILC2s as a source of IL-13 in a mouse model of pulmonary fibrosis. Using an egg-induced pulmonary granuloma model, the authors found that ILC2s numbers increased in IL-25 positive mice and that IL-13 production by ILC2s was a potent driver of pulmonary collagen deposition (52).

However, in certain circumstances, ILC2s were reported to have anti-tumor properties. In a murine lymphoma model, production of IL-33 within the tumor locally enhances the number of ILC2s with potent anti-tumor activity. IL-33 induces ILC2s to secrete a large amount of the CXCR2 ligands CXCL1 and CXCL2. IL-33 also triggers CXCR2 upregulation in tumor cell surface through a dysfunctional angiogenesis leading to ROS production. Tumor cell-specific apoptosis is caused by the interaction between CXCR2 and its ligands, leading to limited tumor growth (53). ILC2s produce IL-5, a cytokine able to stimulate B cells and to recruit and activate eosinophils. IL-5 was found to limit B16 melanoma tumor establishment in mice lungs. IL-25 and IL-33 activated ILC2s were the main source of IL-5 in this model. IL-5 recruited eosinophils, leading to an elevated and prolonged lung eosinophilia anti-tumor activity (54). Interestingly, ILC2s were found at a lower percentage in non-small cell lung carcinoma (NSCLC) patient lung's compared to healthy tissue, suggesting that ILC2s may play a positive role in anti-tumor immune responses in the lung (47).

Overall, although IL-13 production was always found to have a negative impact on the immune response, the consequences of IL-33 or IL-25 on ILC2s in a tumor may vary depending on organs or on the cellular and molecular environment. If ILC2s can enhance immunosuppression along with M-MDSC and Tregs, they can also potentiate eosinophils anti-tumor properties. Additional studies are required to understand key factors involved on the shift of ILC2s from immunosuppression to anti-tumor response.

## ILC3s

Th17 role in cancer progression is controversial (Table 3). This can be partially explained by the importance of the cytokine milieu on Th17 effector functions. Depending on the presence of TGF-β, Th17 can acquire an inflammatory or a regulatory phenotype. Inflammatory Th17 produce IFNγ and IL-17 and exert anti-tumor effects while regulatory Th17 secrete IL-10 and IL-17 and transform ATP into the immunosuppressive molecule adenosine thus sustaining tumor development (67). Likewise, ILC3s, the innate counterpart of Th17, were shown to have both pro and anti-tumor functions. Cell heterogeneity in group 3 ILCs also explains some of the variations observed in the anti-tumor immune response.

**Table 3:** ILC3 and ILCreg phenotypes, functions, and associated cells in solid and hematological cancers.

	**Organism**	**Cancer**	**Effect against cancer**	**Phenotype**	**Responsive to**	**Production**	**Associated cell**	**Observations**	**References**
ILC3	Human	NSCLC	+	NCR+				ILCs located at the edge of TLS	(47)
	Human	NSCLC	+	Lin– CD127+ NKp44+	NKp44 ligand on tumor cells	Production of TNFα, IL-8 and LTαβ	Fibroblast and MSC	Accumulation of ILC3s in lung tissue associated with potential TLS, they upregulate icam vcam on MSC	(47)
	Human	Acute myeloid leukemia	ND	Lin– CD127+ CD117+ CRTH2– NKp46+				Decrease in ILC3 % by 3 times in PBMC, levels back to normal in patients responding to the chemotherapy	(35)
	Human	Breast	–	RORgt+ CD127+ CD3–	CCL21 and CXCL13			Increase in ILC3s % in the tumor linked with an increase in lymph node metastasis	(60)
	Human	Chronic lymphocytic leukemia	ND	Lin– CD127+ CD161+ CRTH2– CD117+ NKp44–				Increase in ILC3s % in PBMC	(36)
	Human	Acute myeloid leukemia	ND	Lin– CD127+ CD117+ CRTH2– NKp46+				Decrease in ILC3 % by 3 times in PBMC, levels back to normal in patients responding to the chemotherapy	(35)
	Mouse	Bacteria induced CRC	–	Lin– IL-22+		Production of IL-22	Epithelial cells and myeloid cells	Accumulation of ILC3s during cancer development, induced epithelial cells proliferation and myeloid cell recruitment	(61)
	Mouse	B16 melanoma	+	NKp46+ NK1,1– CD49b–	IL-12		CD31+ cells	Accumulation of ILC3s in tumor and upregulation of icam vcam on tumor vasculature	(62)
	Mouse	B16 melanoma	+	RORgt+ CCR6+ CCR2+ CCR7+ CCR8+ CXCR5+	IL-12			ILC3s suppress tumor growth and increase CD45 recruitment to the tumor	(63)
	Mouse	4T1 mammary cancer	–	CD3– CD11c– B220– CD127+ CD90.2+ NKp46–	CCL21 and CXCL13		Stromal cells	Increase in ILC3 % in tumor and ILC3s favor lymph node metastasis by inducing the expression of rank and increasing cancer cell motility	(60)
	Mouse	B16 melanoma	–	CD3– CD4+ RORgt+	CCL21		Treg and MDSC	Increased number of ILC3s in tumor	(64)
	Mouse	Duodenal adenoma	–	Thy1+ IL-23R+ NKp46+	IL-23	Production of IL-17		Increased number of ILC3s in duodenal lamina propria	(65)
ILCreg	Human	High grade serous ovarian cancer	–	CD56+ CD3– NKp46+ RORgt–		Production of IL-22		ILCreg limit T cell expansion via NKp46, reducing T cell number and altering their functions	(66)

*ND, not determined; NSCLC, non-small cell lung carcinoma; CRC, colorectal cancer; M-MDSC, monocyte-myeloid derived suppressor cells; PBMC, peripheral blood mononuclear cells; TLS, tertiary lymphoid structure*.

If LTI cells are absent in the organism after birth, some ILC3s may continue to exert some of their functions. Tertiary lymphoid structures (TLS), ectopic lymphoid formations commonly found in NSCLC, are predictors of a favorable clinical outcome (68). NCR+ ILC3s were found located at the edge of tumor associated TLS in patients with NSCLC and a positive correlation between TLS and ILC3s was shown. Moreover, the presence of NCR+ ILC3s within tumor tissues significantly decreased in advanced cancer stages. These NCR+ ILC3s could recognize lung tumor cells via NKp44, triggering a strong production of TNFα. TNFα could activate endothelium, facilitating leukocytes entry in the tumor (47). A similar role was also observed in B16 melanoma mouse model. NKp46+ ILC3s reacted to locally produced IL-12 and induced ICAM and VCAM upregulation leading to an increased leukocyte invasion and tumor suppression (62). In PBMC of patients with acute myeloid leukemia, NCR+ ILC3s number was diminished of about 3 times. Interestingly, this number was significantly increased in patients responding to chemotherapy compared to the number found prior treatment, reaching the values normally observed in healthy individuals.

CCR6+ ILC3s cells were shown to possess anti-tumor functions in certain contexts. The recruitment of immune cells to the tumor is essential to allow a potent anti-tumor immune response. Several studies demonstrated that an important immune infiltrate was a predictor of a good outcome in various cancers (breast, colorectal, ovarian, NSCLC) (69–73). In line with this observation, splenic CCR6+ ILC3s cells can, after activation by IL-12, suppress tumor growth and increase immune cell recruitment (specifically CD8 T cells, NK, and NKT cells as well as activated myeloid cells) in the B16 melanoma mouse model. Recent data showed that these ILCs expressed high levels of CCR2, CCR6, CCR7, CCR8, and CXCR5 suggesting a possible involvement in the formation of TLS (63). In the skin, Notch1 mediated upregulation of TNFα, CCL20 and CXCL13 attracted CCR6+ ILC3s that orchestrated macrophages entry and wound closure through the production of CCL3 (74). This study illustrates the critical role of ILC3s in tissue injury resolution which can be potentially be a major issue in cancer since sometimes this pathology can be seen as a wound that does not heal (75).

However, ILC3s do not always have a beneficial impact on cancer evolution. Bacteria induced colon cancer model shows an accumulation of ILCs where the depletion of IL-22 or CD90 reduced cancer development (61). The authors also showed that CCR6+ ILC3s are the major source of IL-22 in Hh+ (*Helicobacter hepaticus*) driven colorectal cancer, a model in which IL-22 is essential for cancer promotion. In human breast cancer, an increased number of ILC3s correlated with an increase number of lymph node metastasis and an increased percentage of NCR- ILC3s was also found in chronic lymphocytic leukemia patients PBMC (60).

The role of Receptor Activator of NF-κB (RANK)—RANK-ligand (RANKL) interaction in the regulation of CCR6+ ILC3s was recently discovered. ILC3-ILC3 interaction through RANK-RANKL negatively controls production of IL-17 and IL-22 in the intestine (76). This system constitutes an efficient negative feedback loop to avoid an overproduction of ILC3 cytokines. However, another recent study indicated RANK-RANKL interaction lead to an increased cancer cell motility and metastasis formation. In this study, ILC3-stromal cell interaction favored the expression of RANKL on the stromal cell surface and its action on RANK expressing 4T1 breast cancer cells, promoted metastasis development into tumor draining lymph nodes. In this model, NKp46- ILC3s were recruited thanks to CCL21 produced by 4T1 tumor cells (60). CCL21 expression by B16 melanoma cells was also able to attract CD3- CD4+ RORγt LTI cells. In this model, CCL21 was responsible for the establishment of a tolerogenic environment within the tumor bed via the recruitment of Tregs and MDSC. Nevertheless, a direct link between ILC3s recruitment and CCL21 enhanced tumor growth and immune tolerance was not established (64).

Like ILC1s, ILC3s are susceptible to the cytokines they are exposed to, in particular IL-23, which is known to activate ILC3s. Systemic IL-23 expression is sufficient to induce duodenal adenoma development within 4 weeks with an incidence of 100%, independently of exogenous carcinogens. Tumor development was dependent on ILC3s but IL-22 and IL-17 do not seem to be involved in the process (65). The exact mechanism remains to be determined.

A regulatory type of ILCs was recently described in human high-grade serous ovarian cancer. These ILCs produce IL-22 and are NKp46+. They were able to limit tumor infiltrating lymphocytes expansion via the NKp46 expression and were associated with reduced T cell numbers and altered T cell functions. Despite NKp46 expression and IL-22 production, these cells differ from ILC3s since they lack RORγt and display unique properties (66). Patients possessing these regulatory ILCs exhibit a reduced immunosurveillance and have a shorter time to relapse.

## Conclusion and Future Perspectives

As with CD4 T helper cells, ILCs role in anti-tumor immune response is ambiguous. Indeed, ILCs can lead to both pro- and anti-tumor effects, depending on the cytokines and chemokines they are exposed to or on the cells they are in contact with. Moreover, it is difficult to strictly assign a specific effect to a particular subset given ILCs plasticity and phenotypic discrepancies.

Being aware of tumor development mechanisms is crucial and we have only begun to unravel the part played by ILCs. Additionally, it is also essential to understand the consequences of anti-tumor treatments on immune system and their impact on ILCs. For instance, radiotherapy can attract antigen presenting cells, and cytotoxic T cells for a limited period of time (77) and we could hypothesize that ILC3s participate to cytotoxic T cell attraction. Similarly, immunotherapy and more specifically immune checkpoint inhibitors, a currently growing field in oncology, effect on ILCs has never been studied. PD-1 (Programmed cell death 1) can negatively regulate KLRG1+ ILC2s (78) and PD-L1 (programmed death-ligand 1) expressing ILC2s can increase the expression of Gata3 and IL-13 production of PD-1+ Th2 cells both *in vitro* and *in vivo* in worm infection models in mice (79). PD-1 was also observed on ILC3s and could decrease their cytokine production in Human (80). Additionally, in Human breast tumor, CTLA-4 expression was increased in ILC1s and ILC2s and PD-1 expression was augmented in ILC2s (32), rendering interesting the study of the impact of checkpoint inhibitors on ILCs in cancer.

Studies over the last few years provided important insights on the role of ILCs in anti-tumor immune responses but many aspects remain unexplored and numerous additional studies will be required to decipher the details of ILCs involvement in tumor immunity.

## Author Contributions

MB wrote and conceived the manuscript. FG wrote the manuscript.

### Conflict of Interest Statement

The authors declare that the research was conducted in the absence of any commercial or financial relationships that could be construed as a potential conflict of interest.

## Abbreviations

ILCs: innate lymphoid cells
NCR: natural cytotoxicity receptor
NSCLC: non-small cell lung cancer
TLS: tertiary lymphoid structures
LTI: lymphoid tissue inducer
NK: natural killer
CRC: colorectal cancer
PD-1: programmed cell death 1
PD-L1: programmed death-ligand 1
IFNγ: produce Interferon γ
GM-CSF: granulocyte-macrophage colony-stimulating-factor
ICOS: inducible T-cell COStimulator
TGF-β: transforming growth factor-β
TNFα: tumor necrosis factor α
TSLP: thymic stromal lymphopoietin
EOMES: Eomesodermin
CTLA-4: cytotoxic T-lymphocyte–associated antigen 4
PBMC: peripheral blood mononuclear cells
RANK: receptor activator of NF-κB
RANKL: RANK-ligand
PGD2: Prostaglandin D2
M-MDSC: monocytic myeloid derived suppressor cells.
